# Predictors of psychiatric disorders in combat veterans

**DOI:** 10.1186/1471-244X-13-130

**Published:** 2013-05-07

**Authors:** Stephanie Booth-Kewley, Emily A Schmied, Robyn M Highfill-McRoy, Gerald E Larson, Cedric F Garland, Lauretta A Ziajko

**Affiliations:** 1 Behavioral Science and Epidemiology Department, Naval Health Research Center, 140 Sylvester Road, San Diego, CA 92106-3521, USA; 2 Department of Family and Preventive Medicine and Moores UCSD Cancer Center, University of California San Diego, 9500 Gilman Dr, La Jolla, CA 92093-0631, USA; 3 Naval Medical Center, San Diego, 34800 Bob Wilson Dr, San Diego, CA 92134, USA

**Keywords:** Psychiatric disorders, Military populations, Marines, Iraq/Afghanistan wars, Veterans, Combat

## Abstract

**Background:**

Most previous research that has examined mental health among Operation Enduring Freedom and Operation Iraqi Freedom (OEF/OIF) combatants has relied on self-report measures to assess mental health outcomes; few studies have examined predictors of actual mental health diagnoses. The objective of this longitudinal investigation was to identify predictors of psychiatric disorders among Marines who deployed to combat in Iraq and Afghanistan.

**Methods:**

The study sample consisted of 1113 Marines who had deployed to Iraq or Afghanistan. Demographic and psychosocial predictor variables from a survey that all Marines in the sample had completed were studied in relation to subsequent psychiatric diagnoses. Univariate and multivariate logistic regression were used to determine the influence of the predictors on the occurrence of psychiatric disorders.

**Results:**

In a sample of Marines with no previous psychiatric disorder diagnoses, 18% were diagnosed with a new-onset psychiatric disorder. Adjusting for other variables, the strongest predictors of overall psychiatric disorders were female gender, mild traumatic brain injury symptoms, and satisfaction with leadership. Service members who expressed greater satisfaction with leadership were about half as likely to develop a mental disorder as those who were not satisfied. Unique predictors of specific types of mental disorders were also identified.

**Conclusions:**

Overall, the study’s most relevant result was that two potentially modifiable factors, low satisfaction with leadership and low organizational commitment, predicted mental disorder diagnoses in a military sample. Additional research should aim to clarify the nature and impact of these factors on combatant mental health.

## Background

Deployment to combat zones may result in a variety of mental health problems, including posttraumatic stress disorder (PTSD), anxiety, and depression [1,2]. Despite substantial research, factors that increase susceptibility of military members to post-combat mental disorders are not fully understood. Factors suspected to play a role in risk of combat-related mental health disorders include degree of combat exposure, deployment stressors, gender, traumatic brain injury (TBI) symptoms, degree of satisfaction with leadership, unit cohesion, organizational commitment, and positive deployment experiences [1,3-9].

Combat exposure is the factor most consistently associated with mental disorders and symptomatology. Research with Vietnam veterans demonstrated substantial associations between combat exposure and PTSD [10,11]. Similar findings have emerged from studies of Gulf War veterans [12-14] and studies of contemporary combatants in Iraq and Afghanistan [1,6,15].

Another potentially important predictor of mental health problems in military personnel is noncombat deployment stressors [7,16-18]. Examples of these deployment stressors include concerns or problems with family members back home, problems with supervisors, lack of privacy, and boredom. A link between deployment stressors and mental disorders has been demonstrated in Vietnam veterans [11,17,18], Gulf War veterans [18], and in combatants of Operation Enduring Freedom and Operation Iraqi Freedom (OEF/OIF) [5].

Another possible risk factor for mental disorders in combatants is TBI symptoms. Although the nature of the relationship between mild TBI symptoms and mental disorders is unclear, there is substantial overlap between symptoms of mild TBI and symptoms of PTSD [19,20]. A study of OEF/OIF veterans [3] found that those with TBI symptoms had higher rates of PTSD, depression, anxiety, and other disorders. In veterans from prior conflicts (e.g., Vietnam, Gulf War), self-reported head injury has been associated with greater risk of depression [21].

Other factors that may have an impact on mental health in military combatants include leadership and unit cohesion. Little research has examined the effects of military leadership on mental health; however, there is some evidence that positive attitudes toward leadership have a beneficial effect on the mental health of combat-deployed military personnel. Perceptions that leadership is supportive are associated with greater satisfaction with the military, higher morale, and other positive outcomes [22]. A study of deployed U.S. soldiers reported that positive attitudes toward leadership were associated with greater perceived well-being [23]. Castro and McGurk [4] found that combat-deployed personnel who rated their leaders favorably were less likely to screen positive for a mental health problem than those who rated them unfavorably. Positive leadership may buffer the negative effects of stress experienced by deployed military personnel [24].

Unit cohesion may also play an important role in military mental health. Some experts consider unit cohesion to be the most important factor in preventing mental health problems among combatants [25]. Military research [26] clearly demonstrates the importance of cohesion for performance, readiness, well-being, and satisfaction with the military. Unit cohesion may be important for combatants because it may mitigate the effects of stress [27] and reduce the risk of PTSD [6,28].

Organizational commitment may also play a role in service members’ risk for developing mental health problems. Organizational commitment is predictive of reenlistment, job satisfaction, morale, and adjustment to the military [29,30]. Service members with a strong sense of commitment may have a higher level of motivation, leading to greater resilience in the face of setbacks. On theoretical and empirical grounds, it seems plausible that organizational commitment could have a protective effect on mental health.

Positive deployment experiences may have a protective effect on mental health. A study of U.S. military peacekeepers deployed to Kosovo found that positive military experiences (such as feeling that one’s mission is successful) were predictive of postdeployment morale [31]. Another study of U.S. peacekeepers found that positive experiences while on deployment were protective against PTSD [7]. It is also known that the experience of positive emotions can buffer the deleterious effects of stress in civilian populations [32,33]. Thus, it seems plausible that positive experiences during deployment could have a protective effect on mental health.

Although women make up about 14% of military service members deployed in support of OEF/OIF, few studies have examined predictors of psychological health outcomes of women serving in these conflicts. Evidence for gender differences among deployers has been mixed [18,34,35] and many studies have relied on data from previous conflicts (e.g., Vietnam, Gulf War). In addition, little is known about the differential factors affecting the incidence of mental disorders in male and female combatants deployed to OEF/OIF [36]. We examined gender differences in psychological outcomes in the present study.

Most previous research that has examined mental health among OEF/OIF combatants has relied on self-report measures to assess mental health outcomes; few investigations have studied predictors of actual mental health diagnoses. The purpose of this study was to use a prospective design to identify demographic and psychosocial predictors of mental health diagnoses in a sample of Marines who deployed to OEF/OIF. The inclusion of these predictors was based on past theory and research, and it was predicted that all of them would be associated with mental health outcomes. Because few studies have sought to determine if different demographic and psychosocial factors are linked with different mental health disorders, an additional objective was to determine whether different factors predict different types of mental health diagnoses (e.g., mood disorders, PTSD) in a combat-deployed sample.

## Methods

### Overview

The present study was a longitudinal investigation of Marines who participated in a previous study, called the Warfighter Status Survey [37]. The original sample of study participants consisted of 1576 Marines, who were surveyed in 2007 and 2008. To be included as a participant in this earlier study (Warfighter Status Survey), individuals had to be active duty or reservist Marines with at least one prior combat deployment to Iraq or Afghanistan. The study sample for the present, longitudinal study (presented in this paper) consisted of 1113 of the original Warfighter Status Survey participants. The sample for the longitudinal study is smaller than the original Warfighter Status Survey sample (N = 1576), because the longitudinal study excluded Marines who had a previous psychiatric diagnosis on record at the time of survey completion, and also excluded Marines for whom follow-up medical or deployment data could not be obtained.

Almost all of the predictor variables for the present study were based on Marines’ responses to the Warfighter Status Survey (all except for number of combat deployments). The Warfighter Status Survey was completed only once by each participant. Participants in the Warfighter Status Survey were asked for identifying information and for permission to be followed up later in military personnel and medical electronic databases. The primary outcome variables for the study (mental disorder diagnoses) were obtained from an electronic database known as the Career History Archival Medical and Personnel System (CHAMPS). Deployment data were obtained from the Defense Manpower Data Center (DMDC) deployment file. Predictor variables (primarily from the Warfighter Status Survey) were examined in relation to outcome variables obtained from CHAMPS. The deployment data (from DMDC) provided one of the predictors (number of combat deployments).

### Participants and procedure

The longitudinal database for this study was created using three sources: (1) Warfighter Status Survey data; (2) medical data from CHAMPS (data regarding mental disorder diagnoses); and (3) deployment data from the DMDC deployment file. Participants in the Warfighter Status Survey [37] were active duty and reservist Marines with at least one prior deployment to a combat area (*N* = 1576).

Participants were recruited from U.S. Marine Corps bases in Southern California and Okinawa, Japan, and completed self-report surveys in group settings between June 2007 and January 2008. Surveys were not administered immediately after Marines returned from deployment; participants simply had to have participated in at least one prior combat deployment to Iraq or Afghanistan to be eligible for the Warfighter Status Survey. In the announcement inviting Marines to participate in the study, they were assured that confidentiality of their data would be maintained. The overall response rate was 78%. To ensure confidentiality, participants’ data were stored separately from identifiers, and after matching, identifiers were stripped from the data. The participants’ chain of command never had access to any part of their data.

To allow for follow-up, the Warfighter Status Survey requested identifying information (name and social security number) from participants. A more complete description of procedures is provided in Booth-Kewley et al. [37]. All research procedures were approved by the Naval Health Research Center Institutional Review Board (protocol NHRC.2007.0003).

Participants were included in the present study if (1) they were active-duty Marines (not reservists) who completed the Warfighter Status Survey; (2) they provided identifying information; and (3) we were able to find matching data for them in CHAMPS and DMDC data files. These three inclusion criteria resulted in a sample of 1291 participants. Because having a previous psychiatric diagnosis would make an individual more likely to develop another diagnosis after exposure to combat, we excluded an additional *n* = 178 participants who had a pre-existing psychiatric diagnoses on record at the time of Warfighter Status Survey completion. This resulted in a final study sample of 1113 for the current analyses.

### Predictor measures

Most of the predictor measures were obtained from the Warfighter Status Survey by Booth-Kewley et al. [37]. Participants completing the Warfighter Status Survey were asked to answer all survey questions with regard to their most recent combat deployment. The only predictor that was not obtained from the Warfighter Status Survey was total number of career combat deployments, which was extracted from the DMDC deployment file.

Although this survey contained a large number of potential predictors of mental health, only the factors for which there were theoretical reasons to expect an association with mental disorders were selected for this study; these factors are described below.

#### Combat exposure

A combat exposure scale was adapted from one used by the Army Mental Health Advisory Team [23]. The scale consisted of 16 items assessing experiences, such as “receiving incoming artillery, rocket or mortar fire” and “knowing someone seriously injured or killed” (α = .92). Participants indicated how often they experienced each combat exposure using a five-point scale (1 = never to 5 = 10 or more times) during their most recent deployment. An overall combat exposure score was created by summing across all scale items. Level of combat exposure was classified into three groups (low, medium, high) based on the tertile distribution of the combat exposure scale scores.

#### Deployment stressors

Deployment stressors were assessed using an 11-item scale; it was adapted from a similar instrument used by Army researchers [23]. This scale consists of questions about deployment stressors, such as “concerns or problems back home,” “problems with supervisor(s) or chain of command,” and “lack of time off” (α = .89). With their most recent deployment in mind, participants were asked to rate each stressor on a five-point scale (1 = very low to 5 = very high). An overall deployment stressor score was created by summing across all scale items. Deployment stressor level was classified into three groups (low, medium, high) based on the tertile distribution of the scale scores.

#### Unit cohesion

Unit cohesion was measured using a four-item scale previously used in other military research [38]. The items were: “The members of my unit had trust in each other,” “The members of my unit cared about each other,” “The members of my unit supported each other as a team,” and “The members of my unit worked well together to get the job done” (α = .93). Participants rated each item on a five-point scale (1 = not at all true to 5 = completely true). An overall score was created by summing across all items. Unit cohesion was classified into three groups (low, medium, high) based on the tertile distribution of the scale scores.

#### Satisfaction with leadership

Satisfaction with leadership was measured using an eight-item scale that was adapted from a leadership scale developed by Army researchers [23]. The scale assesses various facets of leadership, such as “showed clear thinking and reasonable actions,” and “told Marines when they had done a good job” (α = .94). Participants rated each item on a five-point scale (1 = never to 5 = always). A leadership satisfaction scale score was created by summing across all items. Leadership satisfaction was classified into four groups (low, medium, high, very high) based on the quartile distribution of the scores.

#### Organizational commitment

A four-item organizational commitment scale was developed specifically for this study. The items were adapted from other organizational commitment scales [30,39]. The items were: “I feel emotionally attached to the Marine Corps,” “I feel a strong sense of belonging to the Marine Corps,” “I believe the Marine Corps takes good care of its people,” and “I will always think of myself as a Marine” (α = .87). Participants rated each item on a five-point scale (1 = strongly disagree to 5 = strongly agree). A scale score was created by summing across all items. Organizational commitment was classified into three groups (low, medium, high) based on the tertile distribution of the scores.

#### Mild TBI symptoms

Mild TBI symptoms were assessed using a set of yes/no questions that asked participants whether, during their most recent deployment, they had received an injury to the head that involved (1) being dazed, confused, or “seeing stars,” (2) not remembering the injury, or (3) losing consciousness (knocked out). A participant was considered to have a positive mild TBI screen if any of these three questions elicited a positive response. Hoge et al. [19] used this procedure in their study of mild TBI among soldiers returning from Iraq.

#### Positive deployment experiences

Positive deployment experiences were measured using two items: “Overall, my recent deployment had a positive effect on my life,” and “I feel pride from my accomplishments during my most recent deployment” (α = .72). These items were rated on a five-point scale (1 = strongly disagree to 5 = strongly agree). Positive deployment experiences was classified into three groups (low, medium, high) based on the tertile distribution of the scale scores.

#### Total number of career combat deployments

Total number of career combat deployments was extracted from the DMDC deployment file. These data were dichotomized into two categories: single deployment or multiple deployments.

#### Demographic and military background variables

The questionnaire asked for the following demographic and military information: gender, age, marital status, rank/paygrade, military occupation, education level, race/ethnic background, and whether the participant had deployed with his or her unit or as an individual augmentee on the most recent deployment. Respondents were also asked to provide the dates and locations of their combat deployments.

### Outcome measures

Outcome measures (e.g., psychiatric diagnoses) were obtained from CHAMPS. CHAMPS is an electronic database maintained by Naval Health Research Center in San Diego, California. CHAMPS contains demographic, personnel, and medical information (including psychiatric data) on all active-duty military personnel [40]. The medical records in CHAMPS originate from Standard Inpatient Data Record, Standard Ambulatory Data Record, and Health Care Service Record files via TRICARE Management Activity. These records are generated for military personnel at every inpatient and outpatient medical encounter, except for those that occur within a combat zone, and those that occur outside the military health care system (i.e., visits at civilian facilities).

Psychiatric diagnoses were the primary outcome in this study. Participants were defined as having a psychiatric disorder if they had an outpatient or hospitalization record during the follow-up period that included an *International Classification of Diseases, Ninth Revision, Clinical Modification* diagnostic code (ICD-9-CM) ranging from 290 to 316 (mental disorders), excluding 305.1 (nondependent tobacco use disorder); 307.81 (tension headache); and 310.20 (postconcussive disorder). The observation period was from the time the participant completed the survey (June 2007–January 2008) until December 2010, when CHAMPS records were extracted for this study. The average follow-up time was 30.2 months (standard deviation = 11.5).

### Statistical analysis

Univariate and multivariate logistic regression were used to determine the influence of the predictors on the occurrence of psychiatric disorders. Odds ratios (ORs) and 95% confidence intervals were calculated for each variable. To facilitate ease of interpretation of results, continuous predictor variables (e.g., combat exposure and unit cohesion) were made into categories.

For the multivariate analysis, all variables that were significant in the univariate analysis (*p* < .10) were entered as candidates into the models. Variables were entered into the logistic regression models using a backward stepwise procedure (criteria *p* < .05 for entry and *p* < .10 for removal). Separate models were developed to identify predictors of receiving (1) a psychiatric diagnoses of any kind, (2) a PTSD diagnosis, (2) an anxiety disorder diagnosis, (3) a mood disorder diagnosis, (4) an adjustment disorder diagnosis, or (5) a substance disorder diagnosis. These were the most common diagnoses in the sample.

Pairwise correlations and variance inflation factors did not reveal any substantial collinearity among model variables. Specifically, none of the covariates used in the models correlated with another covariate with a correlation ≥ .48. Statistical analyses were performed using SPSS software, version 18 (SPSS Inc., Chicago, IL).

## Results

The demographic characteristics of the sample are shown in Table 1. The participants were mostly men (96%). The most common ethnic groups were white (59%) and Hispanic (19%). About half the sample had a high school diploma or equivalency degree or a lower level of education (53%). About half of the participants were married (47%). Slightly more than half had experienced one combat deployment during their career (57%); 43% had deployed twice or more. The sample was generally similar to the Marine Corps population on demographics, except that Hispanics were overrepresented in the study sample, and individuals in the lowest paygrades (E1–E3) were somewhat underrepresented in the study sample.

**Table 1:** **Demographic characteristics of study participants, Marines deployed to combat (*N *= 1113), 2007−2010**

**Demographic characteristic**	***n***	**%**
Gender		
Men	1069	96.0
Women	44	4.0
Race/ethnicity		
White, non-Hispanic	656	58.9
Black, non-Hispanic	137	12.3
Hispanic	210	18.9
Other	110	9.9
Education		
High school or less	593	53.3
Some college or college degree	520	46.7
Age, years		
18–21	337	30.3
22–26	436	39.2
≥27	340	30.5
Marital status		
Never married	518	46.5
Married	528	47.4
Divorced	67	6.0
Paygrade/rank		
Enlisted^*^		
E1–E3	274	24.6
E4–E6	615	55.3
E7–E9	121	10.9
Warrant Officer (W1–W5)	34	3.1
Officer (O1–O5)	69	6.2
Deployment status		
Member of deployed unit	947	85.1
Individual augmentee	166	14.9
Total career combat deployments		
1	633	56.9
≥2	480	43.1

^*^E1–E3, Private through lance corporal, *E4–E6*, Corporal through staff sergeant, *E7–E9*, Gunnery sergeant through sergeant major.

### Predictors of overall psychiatric disorders

Of Marines with no previous psychiatric diagnoses at the time of survey completion (*N* = 1113), a total of 18% (*n* = 199) received a psychiatric diagnosis during the observation period. Of the participants who received a postsurvey psychiatric diagnosis, 52% had a single diagnosis (*n* = 103). Comorbid diagnoses were fairly common; 25% of the sample had two diagnoses (*n* = 50), 13% had three diagnoses (*n* = 26), and 10% had four or more diagnoses (*n* = 20). In this sample of 1113, the most common psychiatric diagnoses received during the observation period were anxiety disorders (*n* = 81), mood disorders (*n* = 62), substance use disorders (*n* = 58), adjustment disorders (*n* = 55), and PTSD (*n* = 53).

Logistic regression was used to determine predictors of receiving any psychiatric diagnosis (Table 2). Overall, 12 of the 15 potential predictors of psychiatric diagnosis were statistically significant in the univariate analysis (*p* < .05), and one additional predictor was of borderline significance (*p* < .10). Gender had the strongest univariate association with psychiatric diagnosis (OR = 3.07 for women, *p* < .01). Other variables that had substantial univariate associations with overall psychiatric disorders included satisfaction with leadership, marital status (divorced), mild TBI, combat exposure, and positive deployment experiences.

**Table 2:** **Logistic regression analysis of demographic and psychosocial variables in relation to all psychiatric disorders among Marines deployed to combat (*N *= 1,113), 2007−2010**

	**Univariate**		**Multivariate**	
**Variable**	**OR**	**95% CI**	**OR**	**95% CI**
Gender				
Male (reference)	1.00		1.00	
Female	3.07^**^	1.64–5.75	2.87^**^	1.48–5.57
Education				
High school or less (reference)	1.00		1.00	
Some college or college degree	0.64^**^	0.47–0.87	0.66^*^	0.47–0.93
Marital status				
Married (reference)	1.00		1.00	
Never married	1.07	0.77–1.47	0.91	0.65–1.29
Divorced	2.13^**^	1.20–3.77	1.76^†^	0.96–3.21
Age, years				
18–21 (reference)	1.00		—	
22–26	0.87	0.61–1.24	—	
≥27	0.63^*^	0.42–0.94	—	
Race				
White (reference)	1.00		—	
Non-White	0.89	0.65–1.22	—	
Total career combat deployments				
1 (reference)	1.00		1.00	
≥2	1.46^*^	1.07–1.98	1.45^*^	1.05–2.00
Deployment status				
Member of deployed unit (reference)	1.00		—	
Individual augmentee	1.07	0.70–1.63	—	
Deployment stressors				
Low (reference)	1.00			
Medium	1.27	0.86–1.88	—	
High	1.56^*^	1.06–2.29	—	
Combat exposure				
Low (reference)	1.00		1.00	
Medium	1.73^**^	1.18–2.54	1.64^*^	1.10–2.43
High	1.42^†^	0.95–2.10	1.22	0.79–1.86
Unit cohesion				
Low (reference)	1.00			
Medium	0.66^*^	0.44–0.98	—	
High	0.68^†^	0.46–1.00	—	
Satisfaction with leadership				
Low (reference)	1.00		1.00	
Medium	0.77	0.52–1.15	0.88	0.58–1.33
High	0.59^*^	0.39–0.91	0.75	0.47–1.19
Very high	0.44^**^	0.27–0.73	0.52^*^	0.30–0.89
Positive deployment experiences				
Low (reference)	1.00		1.00	
Medium	0.60^**^	0.42–0.87	0.70^†^	0.47–1.03
High	0.63^*^	0.42–0.93	0.82	0.53–1.28
Mild traumatic brain injury				
No (reference)	1.00		1.00	
Yes	1.78^**^	1.17–2.71	1.85^**^	1.17–2.93
Organizational commitment				
Low (reference)	1.00			
Medium	0.74	0.52–1.06	—	
High	0.62^*^	0.42–0.92	—	

Dashes indicate that variable was not retained in the final model because its association was not statistically significant. *CI*, confidence interval; *OR*, odds ratio. *p < 0.05; **p < 0.01; ^†^*p* < .10.

In the multivariate model, the variables with the strongest associations with any psychiatric diagnosis were female gender, satisfaction with leadership, and mild TBI (Table 2). Women were nearly three times as likely as men to receive a psychiatric diagnosis during the observation period (OR = 2.87, *p* < .01). There was a strong inverse association between satisfaction with leadership and psychiatric diagnosis. Participants in the highest quartile of satisfaction with leadership were about half as likely to develop a mental disorder as those in the lowest quartile (OR = 0.52, *p* < .05). Participants who reported any mild TBI symptoms were nearly twice as likely to develop a psychiatric disorder as those who reported no TBI symptoms (OR = 1.85, *p* < .01).

Other significant predictors of mental disorder diagnosis included education level (more education was protective), marital status (being divorced was associated with the highest risk), total number of career combat deployments (multiple deployments was associated with the highest risk), combat exposure (moderate exposure was associated with the highest risk), and positive deployment experiences (a moderate level was the most protective).

### Predictors of specific psychiatric disorders

Analyses were performed to identify predictors of the more common mental disorder diagnoses in the sample (Table 3). As stated previously, the most common diagnoses were anxiety disorders, mood disorders, substance use disorders, adjustment disorders, and PTSD. Because of heightened interest in PTSD and anxiety disorders in general in military samples, separate analyses were performed to identify predictors of PTSD specifically, and the broader anxiety disorder category (which subsumes PTSD). These two categories are not mutually exclusive; of the 81 participants with an anxiety disorder diagnosis, 53 participants had PTSD. It should also be noted that mood disorder diagnoses in this sample consisted mainly of major depression, dysthmia, and other depression (only 3% had a bipolar disorder).

**Table 3:** **Multivariate logistic regression analysis of demographic and psychosocial variables in relation to specific mental disorder diagnosis among Marines deployed to combat (*N *= 1113), 2007−2010**

	**PTSD**		**Anxiety**		**Mood disorders**		**Adjustment disorders**	
	***n *= 53**		***n *= 81**		***n *= 62**		***n *= 55**	
**Variable**	**OR**	**95% CI**	**OR**	**95% CI**	**OR**	**95% CI**	**OR**	**95% CI**
Gender								
Male (reference)	1.00		1.00		1.00		1.00	
Female	2.06	0.65–6.54	2.57^*^	1.07–6.15	5.68^**^	2.54–12.73	3.09^*^	1.14–8.37
Education								
High school or less (reference)	1.00		1.00		1.00		1.00	
Some college or college degree	0.56†	0.28–1.09	0.58^*^	0.36–0.95	0.77	0.45–1.32	0.54^*^	0.30–0.98
Race								
White (reference)	1.00		1.00		1.00		1.00	
Non-White	0.95	0.52–1.71	0.95	0.59–1.53	1.03	0.61–1.76	0.85	0.49–1.50
Marital status								
Married (reference)	1.00		—		—		—	
Never married	0.40^**^	0.20–0.78	—		—		—	
Divorced	1.17	0.44–3.12	—		—		—	
Age, years								
18–21 (reference)	1.00		—		—		—	
22–26	0.85	0.43–1.71	—		—		—	
≥27	0.37^*^	0.13–0.99	—		—		—	
Total career combat deployments								
1 (reference)	1.00		1.00		1.00	1.00	1.00	
≥2	1.71^†^	0.94–3.11	1.93^**^	1.20–3.09	1.99^*^	1.17–3.40	1.72^†^	0.99–3.00
Combat exposure								
Low (reference)	1.00		—		—		—	
Medium	1.72	0.77–3.81	—		—		—	
High	2.50^*^	1.18–5.30	—		—		—	
Deployment stressors								
Low (reference)	—		1.00		—		—	
Medium	—		1.88^*^	1.00–3.48	—		—	
High	—		1.44	0.75–2.74	—		—	
Unit cohesion								
Low (reference)	1.00		—		—		—	
Medium	0.57	0.28–1.14	—		—		—	
High	0.50^†^	0.24–1.02	—		—		—	
Satisfaction with leadership								
Low (reference)	—		1.00		—		—	
Medium	—		0.81	0.46–1.41	—		—	
High	—		0.38^**^	0.18–0.77	—		—	
Very high	—		0.45^*^	0.21–0.97	—		—	
Positive deployment experiences								
Low (reference)	1.00		—		—		—	
Medium	0.91	0.48–1.74	—		—		—	
High	0.44^†^	0.18–1.06	—		—		—	
Organizational commitment								
Low (reference)	—		—		1.00		1.00	
Medium	—		—		0.58^†^	0.32–1.05	0.73	0.40–1.33
High	—		—		0.42^*^	0.21–0.84	0.37*	0.17–0.82

The demographic variables of gender, education, and race were controlled for in each multivariate model.

The following variables are not presented in the table because they were not significant in any model: deployment status, mild traumatic brain injury symptoms, and mental health care stigma.

Dashes indicate that variable was not retained in the final model because its association was not statistically significant.

CI, confidence interval; OR, odds ratio; PTSD, posttraumatic stress disorder.

**p < .01; *p < .05; †p < .10.

Three variables were significant predictors of PTSD in the multivariate model: combat exposure, age, and marital status (Table 3). Marines who experienced a high level of combat exposure in their most recent deployment were two and a half times as likely to develop PTSD as those who experienced a low level of exposure (OR = 2.50 comparing highest and lowest tertiles, *p* < .05). Older Marines (27 years or older) were much less likely to be diagnosed with PTSD than Marines aged 19–21 years (OR = 0.37, *p* < .05). Marines who had never been married were at reduced risk for PTSD (OR = 0.40 comparing never married and married Marines, *p* < .01). Four additional variables had marginally significant (*p* < .10) associations with PTSD: education, unit cohesion, positive deployment experiences, and total number of career combat deployments. Marines with more education were less likely to be diagnosed with PTSD, as were Marines who reported a high level of unit cohesion and a high level of positive deployment experiences. Marines who had been on multiple deployments were more likely to develop PTSD than those who had deployed only once.

The strongest predictor of anxiety disorders was dissatisfaction with leadership (Table 3). Participants in the highest quartile of satisfaction with leadership had less than half the risk of developing an anxiety disorder as those in the lowest quartile (OR = 0.45, *p* < .05). Other predictors of anxiety disorders (*p* < .05) included female gender, education, number of combat deployments, and deployment stressors. Female participants, those with less education, and those with multiple combat deployments were at increased risk for anxiety disorders. Deployment stressors was also significantly linked with anxiety disorders, but the trend was not linear.

The strongest predictor of mood disorders was gender (Table 3). Female Marines were over five and half times as likely as males to receive a mood disorder diagnosis (OR = 5.68, *p* < .01). Number of combat deployments and low organizational commitment were also significant predictors of mood disorders. Marines who had been on multiple combat deployments in their career were about twice as likely to develop a mood disorder as those who had deployed only once (OR = 1.99, *p* < .05). Marines who reported a high level of organizational commitment were at reduced risk for a mood disorder compared with Marines who reported a low level of organizational commitment (OR = .42 comparing highest and lowest tertiles, *p* < .01).

There were three significant predictors of adjustment disorders: gender, education, and organizational commitment. Female Marines were over three times as likely as male Marines to receive an adjustment disorder diagnosis (OR = 3.09, *p* < .05). Education was protective; Marines who had some college or a college degree were about half as likely to develop an adjustment disorder as those with less education (OR = 0.54, *p* < .05). Organizational commitment was also inversely associated with adjustment disorders (OR = 0.37 comparing highest and lowest tertiles, *p* < .05).

Age was the only significant predictor of substance use disorders in the multivariate logistic model (results not shown). Marines who were 27 years or older were less likely to receive a substance use diagnosis than young Marines (18–21 years old; OR = 0.25; *p* < .01).

## Discussion

This study examined potential predictors of new-onset psychiatric disorders among Marines who deployed to combat in support of OEF/OIF. In a sample of 1113 active-duty Marines with no known previous psychiatric disorders, 18% (*n* = 199) were diagnosed with a new-onset psychiatric disorder during the observation period. Adjusting for other variables, the strongest predictors of overall psychiatric disorders were female gender and mild TBI symptoms, while there was a strong inverse association with satisfaction with leadership.

This study’s finding that female gender was associated with an increased risk of psychiatric disorders is consistent with other military research [41-43]. Goodman et al. [41] found that female gender was an important risk factor for becoming a psychiatric casualty in a sample of U.S. soldiers deployed to Iraq. Similarly, Rundell’s investigation of U.S. military personnel engaged in OEF/OIF [9] found a higher rate of psychiatric evacuation for women than for men. Kehle et al. [43] and Riddle et al. [8] found higher rates of common mental disorders (depression, anxiety, PTSD) among female than male military personnel. Civilian studies have also identified female gender as a risk factor for common mental disorders, such as anxiety and depression [44,45].

Women who deploy to combat zones may be particularly susceptible to psychiatric disorders. These data may be a reflection of the expanding roles of female military members in the current conflicts (OEF/OIF). However, reasons for the elevated levels of mental disorders among female military members are still not well understood. While it is possible that combat exposure has a different effect on women than men, it may be that factors such as sexual harassment, sexual assault, lack of social support, marginalization, and preservice psychosocial history play a role in the gender difference [18,36,46]. In addition, it is possible that military women have a greater propensity than their male counterparts to seek professional help for mental health problems; this is an issue that will need to be addressed in future studies. However, there is evidence that female veterans generally exhibit higher internalizing symptoms (e.g., anxiety and depression) in response to combat, whereas male veterans exhibit greater externalizing symptoms, such as substance use and antisocial behavior [8,47].

We found that veterans who reported at least one symptom of mild TBI were at increased risk for psychiatric disorders. Although this finding is consistent with other military studies [3,19,21], this topic deserves additional attention. Carlson and colleagues [3] found that nearly half of the OEF/OIF war veterans screened for TBI in their sample had at least one psychiatric diagnosis, with PTSD and depression being the most common. Elevated rates of psychiatric disorders have also been found in civilians with a history of TBI [48]. The nature of our data does not allow us to draw conclusions regarding causality with regard to mild TBI symptoms and mental disorders. Prospective, longitudinal research involving precise assessment of head injury events, TBI symptoms, psychiatric symptoms, preexisting psychiatric conditions and outcomes will be needed to determine the nature of this association.

A unique finding of this study was the association between satisfaction with leadership and mental disorders. Adjusting for other variables, service members who expressed a high level of satisfaction with leadership were about half as likely to develop a mental disorder as those who were not satisfied. This finding suggests that for military personnel who deploy to combat, good leadership may be a key protective factor against psychiatric problems. This finding is consistent with research showing that positive leadership has a beneficial effect on the mental health of combatants [4,22,23]. To our knowledge, the present study is the first prospective military study to link leadership dissatisfaction with diagnosed mental disorders. An implication of these findings is the need for the military to continue to develop programs to improve leadership.

Consistent with previous research [6,42], Marines who experienced a higher level of combat exposure, and younger Marines were at increased risk for PTSD. Also, Marines who had never been married were at reduced risk for PTSD. Marital status findings in past military research have not been consistent. Some studies have found that divorced individuals are at higher risk for PTSD [49,50]; others have found minimal or no associations between marital status and PTSD [51,52].

Similar to its inverse relationship with general mental disorders, satisfaction with leadership had a strong inverse association with anxiety disorders. Service members who expressed a high level of satisfaction with leadership were less than half as likely to develop an anxiety disorder as those who were not satisfied. Our finding that lower education was a risk factor for anxiety disorders is consistent with both military research [53,54] and civilian research [55] linking low education with PTSD and other psychiatric problems.

Results for mood disorders and adjustment disorders were similar. The key factors associated with mood disorders were female gender, number of combat deployments, and organizational commitment. The key factors associated with adjustment disorders were female gender, lower education level, and organizational commitment. Service members who expressed a high level of organizational commitment were less than half as likely to develop a mood disorder or an adjustment disorder as those with low organizational commitment. These results are consistent with research demonstrating that organizational commitment is associated with reenlistment, job satisfaction, morale, and adjustment to the military [29,30]. Having a strong sense of belonging to the military organization and strongly internalized military values may help to foster psychological resilience in the face of deployment stress.

Partial support was found for the hypothesis that deployment stressors would predict overall psychiatric diagnoses. Deployment stressors had a significant univariate association with psychiatric outcomes (nonsignificant in the multivariate model), and was predictive of anxiety disorders in the multivariate model. These findings are consistent with previous research finding relationships between deployment stressors and PTSD symptom scales [5,11]. To our knowledge, this is the first study to find an association between deployment stressors and diagnosed anxiety disorders.

The present study had limitations. One limitation is that our sample included only a small number of women, and these women may not be representative of the female Marine Corps population. Most of the predictor variables used in the study were based on self-report, with its associated limitations (e.g., response bias and socially desirable responding). Another limitation relates to the fact that the surveys asked for identifying information. Although confidentiality was assured, it is likely that some degree of underreporting occurred. Also, the military database from which mental disorder diagnoses were drawn did not contain information about diagnoses assigned within the theater of operations or diagnoses assigned outside the military health care system.

One other limitation of the study is that the number of psychiatric diagnoses in the sample was relatively small, making it likely that we lacked sufficient power to detect small effects. Another notable limitation relates to our use of military medical records for the mental disorder outcome data. Combat veterans in our sample who had a mental disorder but who never sought help would have been counted as not having a psychiatric diagnosis, thus adding error to the data. It is likely that this underreporting of common mental disorders (e.g., anxiety, mood disorders) would have lead to a reduction in the effect sizes found in this study, compared with true effects sizes that would have been found if all cases of mental disorders were known. In other words, the results reported in the present study are probably an underestimation of the true effect sizes.

## Conclusions

In terms of translating research into practice, this study’s most relevant results were that two potentially modifiable factors, dissatisfaction with leadership and low organizational commitment, were predictive of psychiatric diagnoses in a military combatant sample. To our knowledge, this is the first study to find predictive associations between these two organizational factors and psychiatric outcomes. This study adds to the research literature because few prospective studies have examined predictors of psychiatric diagnoses in contemporary combat-deployed samples. Another unique feature of this study was the availability of a wide range of predictor variables for a cohort of military members who were initially free of a diagnosis of a mental disorder while in the military. The available data allowed us to evaluate a broad range of potential predictors of mental health problems. Additional research should aim to clarify the nature and impact of these factors on combatant mental health. It is also recommended that the military continue to develop programs to strengthen leadership and to foster greater organizational commitment among its members, since both factors may lead to improved psychological health.

## Abbreviations

CHAMPS: Career History Archival Medical and Personnel System; DMDC: Defense Manpower Data Center; ICD-9-CM: International Classification of Diseases Ninth Revision, Clinical Modification; MHAT: Mental Health Advisory Team; OEF/OIF: Operation Enduring Freedom and Operation Iraqi Freedom; OR: Odds Ratio; PTSD: Posttraumatic stress disorder; SPSS: Statistical Package for the Social Sciences; TBI: Traumatic brain injury.

## Competing interests

The authors declare that they have no competing interests.

## Authors’ contributions

SBK conceived of the study, developed the study design, performed statistical analysis, and drafted the manuscript. ES assisted with study design, performed data management and statistical analysis, and assisted in drafting the manuscript. RMH assisted with study design and statistical analysis, and made revisions to the manuscript. GEL assisted with study design and interpretation of results, and made revisions to the manuscript. CFG consulted on the study methodology and statistical approach, and assisted in drafting the manuscript. LAZ assisted with study design and interpretation of results, and assisted in drafting the manuscript. All authors read and approved the final manuscript.

## Pre-publication history

The pre-publication history for this paper can be accessed here:

http://www.biomedcentral.com/1471-244X/13/130/prepub
